# Social determinants of health pave the path to maternal deaths in rural Sri Lanka: reflections from social autopsies

**DOI:** 10.1186/s12978-022-01527-2

**Published:** 2022-12-05

**Authors:** Lasandha Irangani, Indika Ruwan Prasanna, Sajaan Praveena Gunarathne, Sandaru Hasaranga Shanthapriya, Nuwan Darshana Wickramasinghe, Suneth Buddhika Agampodi, Thilini Chanchala Agampodi

**Affiliations:** 1 Department of Humanities, Faculty of Social Sciences and Humanities, Rajarata University of Sri Lanka, Mihintale, 50300 Sri Lanka; 2 Department of Economics, Faculty of Social Sciences and Humanities, Rajarata University of Sri Lanka, Mihintale, 50300 Sri Lanka; 3 Department of Community Medicine, Faculty of Medicine and Allied Sciences, Rajarata University of Sri Lanka, Saliyapura, 50008 Sri Lanka

**Keywords:** Maternal death, Social autopsy, Social determinants of health, Sri Lanka

## Abstract

**Background:**

Ending preventable maternal deaths remains a challenge in low- and middle-income countries (LMICs). Society perceived causes and real-life observations can reveal the intangible causes of maternal deaths irrespective of formal maternal death investigations. This study reports complex patterns in which social determinants act towards paving the path to maternal deaths in a rural Sri Lankan setting.

**Methods:**

We conducted social autopsies for 15/18 maternal deaths (in two consecutive years during the past decade) in district A (pseudonymized). In-depth interviews of 43 respondents and observations were recorded in the same field sites. During thematic analysis, identified themes were further classified according to the World Health Organization framework for social determinants of health (SDH). The patterns between themes and clustering of social determinants based on the type of maternal deaths were analyzed using mixed methods.

**Results:**

Discernable social causes underpinned 12 out of 15 maternal deaths. Extreme poverty, low educational level, gender inequity, and elementary or below-level occupations of the husband were the characteristic structural determinants of most deceased families. Social isolation was the commonest leading cause manifesting as a reason for many other social factors and resulted in poor social support paving the path to most maternal deaths. A core set of poverty, social isolation, and poor social support acted together with alcohol usage, and violence leading to suicides. These core determinants mediating through neglected self-health care led to delay in health-seeking. Deficits in quality of care and neglect were noted at health institutions and the field.

**Conclusion:**

Social autopsies of maternal deaths revealed complex social issues and social determinants of health leading to maternal deaths in Sri Lanka, indicating the need for a socially sensitive health system.

## Plain English summary

Many women in low and middle-income countries die during pregnancy, childbirth, or postpartum due to preventable factors. Routine inquiries into these deaths conducted by health personnel focus mainly on problems in health systems, neglecting social causes. We explored how social factors paved the path to maternal deaths inquiring the immediate community. We conducted 43 interviews with close persons and observations of 15/18 deceased women within 2 consecutive years, in district A (pseudonymized) in Sri Lanka. We included all deaths that occurred in the hospital as well as the field during the study period. The patterns of social causes based on the type of maternal deaths were analyzed using mixed methods. Extreme poverty, low educational level, gender inequity, and elementary or below-level occupations of the husband were common in most deceased families. Social isolation was the commonest leading cause and resulted in poor social support making women helpless. We observe that poverty, social isolation, and poor social support at family and neighborhood level acted together with alcohol usage and violence, leading to suicides and to delay in health-seeking. Deficits in quality of health care and negligence were noted in hospitals and in field care provision. We conclude that it is of prime importance to identify social causes leading to deaths of women during pregnancy and postpartum, as prevention of these is possible through simple multisectoral interventions. The health system needs to be integrated with social interventions and systems at community level to identify women at risk.

## Background

Reducing maternal mortality is a challenge for health care systems, especially in low- and middle-income countries (LMIC). The United Nations have re-set its target [1] in the Sustainable Development Goals (SDGs) to reduce the maternal mortality ratio to less than 70 per 100,000 live births by 2030 [2]. Focusing on its motto, *“leave no one behind,”* SDGs emphasize the need to safeguard equity in health across populations [3] to further reduce maternal mortality. Identifying the varity of factors and their interplay leading to maternal deaths at contextual level is essential in further reduction of maternal deaths.

The International Classification of Diseases for Maternal Mortality (ICD-MM) classifies maternal deaths using direct and indirect biomedical causes [4]. Most deaths in LMICs occur due to direct obstetric complications during pregnancy, childbirth, or postpartum due to omissions and incorrect treatment. An indirect death occurs due to an exacerbated pre-existing disease. The ICD-MM classification included maternal suicides as direct obstetric deaths, a decision made to overcome the de-recognition of maternal deaths due to underlying psychosocial causes [4, 5]. Still the bio-medical focus on this classification hinders identifying social causes that push women towards adverse scenarios.

The global emphasis on Social Determinants of Health (SDH) [6] has led to the identification of social risk factors for maternal health [7, 8]. Many structural determinants; poor literacy level of the household members [9], higher maternal age [10], low level of education of the women [11, 12], poor economic condition [13], cultural factors including gender, family, caste, class are risk factors of maternal mortality. Poor access to health services [14], low social support, abuse and violence [15], and nutritional inadequacies [16] act as intermediary SDH giving rise to inequities in maternal survival.

Society perceived factors of maternal deaths could be intangible at the time of the routine maternal death investigations which are conducted based on the three delays; deciding to seek health, reaching a medical facility, and in care provision in the facility [17]. This invariably occurs in the scenario of focusing the delays of health care service provision during maternal death investigation neglecting the social causes. Social autopsy is a process utilized to identify the intangible factors ingrained in the context as perceived by the society in a situation of a death. Findings of social autopsies related to maternal deaths may inform health policy at the global, national, and subnational levels [18]. It would shed light to reinforce vigilance on social factors and incorporate appropriate preventive mechanisms in order to achieve SDGs.

Sri Lanka is a lower middle income country which is in the fourth stage of obstetric transition [19]. Accordingly, Sri Lanka’s maternal mortality rates are lower than that of other developing countries (30.2/100,000 Live births). This achievement is attributed to rapid health system development during the past century as well as to the provision of free education and free health services by the government [20]. The cultural factors that prioritize mothers and children preserving comparative gender equity is also mentioned as a contributory mechanism that have led to success. According to the national statistics causes of maternal deaths have shifted from direct obstetric causes to indirect causes during the past decade. Apart from obstetric hemorrhage and amniotic fluid embolism which are among the direct obstetric causes of maternal deaths in Sri Lanka, indirect causes such as heart disease in pregnancy, respiratory diseases (Mainly H1N1influenza epidemics exerting time to time since 2015) and dengue have been among the leading causes of classified maternal deaths in the recent past [21].

Suicides, self-harm and suicidal ideation of reproductive age females is a huge problem in Sri Lanka [22]. It is well known that maternal suicides have been rising during the past two decades [23]. Cultural tendencies of impulsive self-harm attempts, poverty and use of alcohol in households and the availability of pesticides [24] which was considered as a main mode of self-harm in agricultural communities in Sri Lanka [25, 26]. Evidence indicate that if the ICD-MM was applied in counting the maternal deaths, maternal suicides would be the single leading cause of maternal deaths in certain provinces of the country [27]. However, majority of reported maternal deaths that occur due to impulsive behaviors are taken up as incidental deaths and not included in maternal mortality estimates in the country. Nevertheless, all suicidal deaths are reviewed and the country is in the process of finding solutions for this problem. Identification of social determinants that lead to impulsive attempts of self-harm and suicide is detrimental at this stage.

Sri Lanka has an efficient maternal death surveillance mechanism which was initiated in 1981 and further streamlined in 1995 [26, 28]. This system includes consideration of all deaths that are reported as maternal deaths and consists of institutional and field investigations which include verbal autopsy and focus on the related documents (conducted within 14 days of a maternal death). The Surveillance system reviews all maternal deaths at regional and National level comprised by a team of experts on public health from that Family Health Bureau, Ministry of Health, Sri Lanka and the relevant colleges (Obstetrics and gynecology, Psychiatry). The effort is assimilated to learning the lessons and appropriate mechanisms are implemented to overcome the delays at National, regional, institutional and at relevant field level. However, despite the effort further reduction of maternal deaths has been a challenge. We hypothesize that one reason for this could be the lack of understanding of social determinants that function at ground level in facilitating maternal deaths, as including social autopsies in to the maternal death investigation procedure would be difficult. The maternal death investigation which is conducted by medical professionals may miss the underlying social factors as it is based on identifying the three delays that would cause maternal deaths [17] and the health service oriented causes for the three delays.

Evidence indicates that further reduction of maternal deaths in Sri Lanka can be achieved by identifying the social and institutional factors that affect maternal deaths [27]. This community-based qualitative study simulating social autopsies was designed to explore those intangible causes and complex social issues linked to maternal deaths.

## Methods

We conducted social autopsies of 15 maternal deaths which occurred in two consecutive years during the recent past in district A (pseudonymized), Sri Lanka using in-depth interviews and observations (The district and the year of maternal deaths are not included to safeguard confidentiality of participants). We report the study findings according to the standards for reporting qualitative research (SRQR) guidelines.

### Study design

Due to the nature of the phenomenon studied, we have adopted a qualitative, case study-based methodology which supports narrowing down a broad research field into a researchable topic [29]. It provides a more interpretive understanding of a particular scenario or a problem. As this study deals with sensitive data where privacy and confidentiality are significant concerns, we have hidden the information which reveals the identity of the respondent.

### Study setting

We conducted the study in a rural setting in a selected district in Sri Lanka. This district was selected as the particular study was conducted as a part of a larger population-based study which took place in the specific district. The district resembles predominantly rural community in Sri Lanka (70% of the Sri Lankan population belongs to rural sector). The district comprises an agriculture-based economy. The median household income is around United States Dollar (USD) 208.6 per month [30]. The ethnic breakdown of the population is represented as Sinhalese 90.7%, Tamil 0.8%, and Sri Lankan Moor 8.1% [31]. In the Sri Lankan maternal health system, all pregnant women are routinely registered by the area of public health midwife to provide care. The Public health midwives provide routine antenatal and postpartum care in the field assisting the medical officers in charge in risk assessment, monitoring maternal and fetal wellbeing and promotion of health. The antenatal care is delivered at field clinics and as domiciliary care by the PHM. The antenatal care coverage of the district is 96%, and 98% of the childbirths take place in health institutions [32]. In 2016, 90% of females in the district had entered at least secondary level education [33]. The district health statistics represent the average Sri Lankan health statistics [32]. However, according to the previous studies suicides remain as the single leading cause of maternal deaths in the district [27].

### Study sample

The study sample consists of close relatives and neighbors of maternal deaths in two consecutive years (pseudonymized) in district A. Eighteen maternal deaths have occurred, and 15 were included in this study based on availability of the spouse/family members or any other related person of the deceased women in the study area at the time of interview conducted. For each death, two or more participants, including members of the deceased pregnant or postpartum women’s family (the husband, mother, father, or another family member of the deceased woman) or an identified close neighbor, were selected (Table 1). The exception was case 11, where no relatives of the deceased women were residing in the area by the time of the interviews.

**Table 1:** Description of the relationship of respondents with the deceased women

Maternal death (case)	Respondents	Relationship with the deceased woman	Duration of the relationship
1	R1	Husband’s elder brother’s daughter	3–4 years
	R2	Husband’s younger brother	3–4 years
	R3	Husband’s younger brother’s wife	3–4 years
2	R4	Husband	Eight years of marriage
	R5	Mother	From birth
	R6	Stepfather	From birth
	R7	Deceased women’s mother’s elder sister	From birth
	R8	Deceased woman’s mother’s elder sister’s husband	From birth
3	R9	Husband	Four years
	R10	Husband’s mother	Four years
	R11	Husband’s father	Four years
	R12	Son (Deceased woman’s 1st marriage)	From birth
	R13	Close neighbor 1	Four years
	R14	Close neighbor 2	Four years
4	R15	Husband’s mother	4–5 years
	R16	Close neighbor	4–5 years
5	R17	Husband	Three years of marriage
	R18	Close neighbor	From birth
6	R19	Husband	Three years
	R20	Husband’s mother’s elder sister	Three years
**7**	R21	Husband	Unmarried relationship for nine years and one year of marriage
	R22	Husband’s mother	10 years
	R23	Father	From birth
8	R24	Mother	From birth
	R25	Father	From birth
	R26	Sister	From birth
	R27	Close neighbor	15 years
9	R28	Husband	10 years of marriage
	R29	Husband’s elder and younger sisters	10–11 years
	R30	Close neighbor	10–11
10	R31	Husband	Five months of marriage
	R32	Husband’s mother	One year
11	R33	Close neighbor	Less than one year
12	R34	Father	From birth
	R35	Husband	One year of marriage
13	R36	Father	From birth
	R37	Husband’s sister	4–5 years
	R38	Close neighbor	Seven years
14	R39	Husband’s mother	1–2 years
	R40	Close friend	1–2 years
15	R41	Husband’s mother	1–2 years
	R42	Mother	From birth
	R43	Sister	From birth

### Recruitment of study participants

We collected basic details of maternal deaths reported in the concerned years from the district health authorities with ethical and administrative clearance. Before recruitment, the participants were informed of the study’s aims. We obtained verbal consent from the participants. Recruitments took place in a location convenient for the participants in their villages.

Selection of participants included a two-step approach. One or more close family members were recruited at the initial stage for each maternal death. After interviewing them for each case an additional suitable participant (neighbor/relative or friend) who could provide information (considering the narration) was selected purposefully with view of triangulation. The third participant was determined while interviewing with family members, and in some specific cases, such as suicides, discussing with a few village members. Selecting this third respondent was based on the information revealed during the interview of the first and second respondents.

Even though, the plan was to conduct three interviews per case, for three cases (Case 2, 3 and 8) we had to do more than three interviews as we felt more information needed to be reveled for such cases. Also, for seven cases (Case 4, 5, 6, 10, 11, 12, and 14), we had to do less than three interviews as lack of related persons to the deceased women at the time of the interview or reluctant to be participated.

Priority was given to building rapport and trust with the interviewers. Study was explained to the participants.

### Interviewers of social autopsies and study instrument

A social science expert proficient in qualitative interview techniques held the interviews, and a trained research assistant took notes. The investigators with a medical background purposefully avoided the interviews concerning the information and interviewer biases anticipated in the topic of concern. Hence the investigators’ position was not medically oriented. The interviewer guide was prepared using standard guidelines [34] and kept open to follow information relevant to each interview using appropriate probes. The interview guide mainly consisted of open-ended questions. All the interviews were conducted in Sinhalese language.

### Data collection

We conducted in-depth interviews on a date and at a convenient place in the key informant’s house. The interviewer maintained a position between “pollster” and “prober” [35]. We held neutral body language as much as possible to allow free conversation. When the participants had complaints, critical reflections on health systems and society, expressed emotions, we listened empathically and showed respect [36]. Each interview lasted for 45 to 60 min. Observations were documented by the investigators visiting the field site. All interviews were tape-recorded with the participants’ permission.

### Data management and analysis

Recordings were transcribed within one month, and each maternal death was given an identification number. All files were stored securely for each maternal death in a soft and hard copy file in a password-protected computer and locked cupboard, respectively, in a location only accessible to the research team.

We used the thematic analysis [37] as we expected to systematically identify the social causes underpinning maternal deaths and the patterns between these social causes. Independent coding was performed by two investigators trained in qualitative research. The coding schemes were developed with consensus. The steps in the thematic analysis included initial coding, coding scheme development, complete coding, identifying sub-themes and themes, and identifying the associations between different themes. Data triangulation from family members of the deceased pregnant/postpartum women and close neighbors was performed to understand the complex social issues.

Following the first coding round, the investigators, comprising social sciences and public health expertise, held several discussions to review the themes and sub-themes identified. They considered field observations of investigators who visited the field site and neighborhoods, the verbatim narratives of the respondents, and the related medical and obstetric circumstances. The possibility of non-reported issues in the transcripts and the ambiguous aspects of the reported issues were discussed in detail and agreed upon with the consensus of all investigators.

According to the WHO framework of SDH inequities [38], the themes identified were further categorized. Independent of this classification, we identified specific clustering patterns of social causes related to the type of death or delay. The identified patterns were tested using Principal Component Analysis (PCA) [39].

### Quality assurance of data

We adopted currently accepted guidelines for designing the tools and collecting data. Data analysis methods were accomplished by paying attention to the transparency and rigor of the procedures. We performed triangulation by collecting data from different individuals on the same case and combining interview data with observation field notes. A summary of the opinions and ideas of the participant as perceived by the interviewer was expressed at the end of the in-depth interviews for agreement to safeguard respondent validation. We explained that the study was not conducted for legal or faultfinding purposes and assured them that their family-centered issues would not be judged or commented on to preserve information bias. We were vigilant on positional reflexivity and adopted diary memos to document incidents referring to such situations.

### Ethical considerations

Ethical clearance was obtained from the Ethics Review Committee, Faculty of Medicine and Allied Sciences, Rajarata University of Sri Lanka. The district and the participants were pseudonymized. After obtaining verbal consent for visiting the residence and being willing to participate in the interviews, we explained the importance of the study and study aims. It was made clear that the interviews are conducted by experts, not in the medical field, and there will be no comptonization of the medical services they receive due to their information. In instances where the participants felt psychologically disturbed due to the interviews, the investigators stayed with them and counseled and empathized (the team conducting the interviews had counseling experts). If a participant needed further psychological support, a mechanism was established to refer them to specialized care. The detailed discussions and recoding during analysis prevented false interpretations and delivered a comprehensive approach to decision-making using qualitative data.

## Results

We conducted 43 in-depth interviews (Table 2) for 15 out of 18 maternal deaths (representing two consecutive years) and prepared 15 field notes for each case. We could not contact anyone to get the information related to the other three deaths (probably due to family members leaving the area).

### Socio-demographic profile of the deceased women in the sample

The mean age of the deceased women was 27.9 (range 24–31.5) years. The details are presented in Table 2. One deceased women had no schooling, four had below primary education, and five studied up to General Certificate of Education (GCE) ordinary level. Two women had degree-level qualifications. Most deceased women were housewives who were not engaged in any income generation activity. According to the International Standard Classification of Occupations (ISCO) [40] majority of husbands had only elementary level occupations (people from armed forces, fishermen, farmers and laborers) of skill level one.

**Table 2:** Socio-demographic information of the deceased women and the characteristics of maternal deaths

Characteristics		n
Marital status at the time of the death	Married	12
	Unmarried	3
The age difference between deceased women and their husbands	Age gap < 15 years	10
	Age gap > 15 years	3
	Husbands age < wife’s age	2
Education level	No schooling	1
	Below primary education	4
	Up to GCE O/L	5
	Degree level	2
	No data	3
Employment status of the deceased women according to ISCO	Housewives (unemployed)	11
	Skill level 2 in the government sector*	2
	Skill level 1—elementary occupation	2
Employment status of the husband according to ISCO	Skill level 1—armed forces	3
	Skill level 1—elementary occupations	8
	Skill level 2 in the government sector—clerical support workers	2
	Skill level 2 in the private sector—clerical support worker	2
Family type when the death occurred	Nuclear family	8
	Extended family	7
Cause of maternal death	Maternal suicide	4
	Direct obstetric causes	2
	Indirect causes	6
	Under investigation/cause inconclusive	3
Time of the death	During antenatal period	8
	Post-abortion	2
	Postpartum	5
Status of the child	Survived	6
	Not survived	9

^*^Exact occupation not mentioned to preserve the confidentiality

### Characteristics of maternal deaths

The majority of the deaths (n = 8) occurred during the antenatal period. Two deaths occurred post-abortion, and five occurred in the postpartum period. Maternal suicides were the single leading cause of 26.7% (n = 4) deaths. The mode of suicide was hanging (two deaths), followed by agrochemical poisoning and burn. Two maternal deaths occurred due to direct obstetric causes, ruptured ectopic and uterine rupture. The identified causes of the rest of the maternal deaths were indirect causes: H1N1 (n = 3), myocarditis (n = 1), meningoencephalitis (n = 1), and septicemia due to cellulitis (n = 1). Three deaths were inconclusive in cause at the district maternal mortality review. During the social autopsies conducted, community members’ perceptions were directed to puerperal sepsis, severe asthma, and an illness representing fever being the possible reasons for the deaths of particular women. Three maternal deaths were directly related to medical negligence, as perceived by the community members.

### Social determinants of health underlying maternal deaths

Structural, intermediary, and health system-related factors (The three major themes) acted at some point, paving the path to maternal deaths. We also identified how these determinants would cluster and their relationship to specific types of maternal deaths and delays during the analysis. We present the results under the themes identified and then elaborate the patterns of causes of maternal deaths.

#### Individual structural social determinants of health underpinning maternal deaths

Individual and family level structural determinants, such as poor educational status of the deceased woman or the family members, poverty, gender inequity, and marital instability, underpinned maternal deaths.

##### Educational deprivation

Society felt inadequate education would prevent a pregnant woman from attending routine clinics, abiding with the health advice given, and understanding the basic health needs during the health care seeking process. In eight families, either the deceased woman or the husband or both had very low education levels.

Social autopsies of C3, C6, and C9 directly suggested illiteracy as a root cause of the death. *“She had not been to the maternal clinic for nine months….”* (C9: R29). *“They are illiterate people. The midwife didn’t know she was pregnant. She did not even go to the antenatal clinic”* C9 (R30).

The husband (C9:R28) has never attended school. The interview revealed that he was unaware of his wife’s pregnancy until a week before the childbirth and death. She has kept her condition a secret, avoiding antenatal clinic visits and public health midwife (PHM). The interview suggested that this woman has encountered a medically contraindicated pregnancy. *“She told us that she was pregnant previously on one occasion, but it was aborted. She said the doctors had informed her not to conceive due to severe diabetes”* (C9: R30).

Findings suggest that poor education can interfere with managing own health conditions, avoiding medically-risk pregnancies, making appropriate health-seeking decisions, and understanding individuals’ health outcomes.

##### Poverty and poor living conditions

Many households that we visited had poor living conditions. We witnessed the true nature of poverty and poor living standards during the field visits. *“There was no one at the house [meant as that] of the deceased woman (C1). After completing the interviews with the deceased woman’s in-laws, we saw another abandoned house (small in size, built-in mud, and dilapidated at the moment) just behind the house where we had interviews. We asked about it from the relatives, and they told us that the deceased women and her husband were living in that old house at the time of the death. I felt it was not a kind of a place to live for a pregnant woman; I could imagine the family’s poverty during the pregnancy”* (C1: Field Note).

Household economic issues during the pregnancy led women to work in the field to earn a living. *“She was going for daily wage heavy labor while she was pregnant. My wife helped them continue their livelihoods and acquire daily essentials at times. That woman lived with numerous economic issues…carrying a child inside, she went to work in the field to obtain a daily wage”* (C13:R38).

The relatives and neighbors emphasized the life-threatening poor living conditions of family members created by extreme poverty. *“That family had many problems. Previously they were not living here. They were isolated from the society and lived in the middle of that jungle…”* (C9:R30). C9 (R29) indicated the severity of the occupation of the deceased woman’s husband, who was a fisherman. “*His occupation was life-threatening. There are crocodiles in those tanks where he works. I always think about what will happen to his children if he meets with an accident”* (C9:R29).

We were convinced that the family would not prioritize their health in such poverty and poor living conditions and may refrain from timely health-seeking.

##### Gender inequity

Society dictated male dominance in decision-making in rural areas. This took away the autonomy of the spouse regardless of education level. “*In the first six months of pregnancy, her husband treated her well. But after knowing her fetus has anomalies, her husband insisted that she abort the child. Eventually, she gave in to her husband’s demand with great dismay ending up with* *severe depression”.* (C14:R40).

In a situation where it was not clear whether the death was a suicide or a homicide, we observed that the gender-related power differences were high as to assimilate false interpretations even at the funeral. *“She told me her husband was having another affair. When she asked about it, he fought with her…. She is not the type of person who would easily give in to suicidal thoughts. Her husband made her feel that way. Even in the post-mortem, they said that she had been beaten brutally…While the body was being embalmed, we saw her husband supplying cosmetics to cover her bluish and puffed-up face”* (C14: R40).

Sometimes, gender inequities in the division of labor led women to engage in heavy work even during the postpartum period. *“After four days of her delivery, she carried a water pot in one hand and the newborn in the other hand. She was carrying water to bathe her husband…, she drew water from the well for her husband to bathe even four days after her delivery, and if not, her husband would beat her. She had no help from her husband….”* (C8:R27).

##### Poor marital cohesion

Poor marital cohesion was noted in six deceased families. In some families, it was confusing for the investigators to identify the actual relationships of family members. The adultery behaviors of both partners was criticized by the community leading to social isolation. *“I felt neighbors around this deceased woman’s house were unwilling to provide information about her. It was challenging for us to find a participant to get the information about the death. Almost all we talked were hesitant to answer about the deceased woman”* (C11: Field Note).

The neighbors looked down on such families. *“This woman had several other husbands before this (extra-marital affairs). She abandoned the two children from her previous marriage and came here with a new husband. We do not know if she was divorced or not. She had been abroad several times to work as a housemaid and had extra-marital affairs there too. So we doubt she had a good family life. Once, she had an abortion as well… There were a lot of family problems* (C11: R33).

Marital disputes resulted in depression and social isolation. *“She and her husband were separated after her first delivery, and they joined again when she was pregnant for the second time. Their family life was full of fights and quarrels. Her husband had a casual affair with another woman next door. When the fights got critical, she went to her mother’s house. Her mother knew about this situation and encouraged her to get a divorce. But she refused it. Eventually, her mother did not care much for her either. She [deceased woman] was very introverted and quiet! She would stay quiet even when she was hungry, and she didn’t say anything.”* (C2: R7). Social isolation created by this family rejection situation and poor maternal responsiveness of her aesthetes worsened her depression.

#### Social isolation, lack of social cohesion, and support

Social cohesion and social capital are considered as a central determinant of health equity indicating to represent both structural and intermediary social determinants of health [41] Social isolation is a resulting manifestation of lack of social cohesion and capital. Social isolation was a factor seen common in 12/15 families. In some families, the isolation was readily visible. Social isolation manifested as an outcome of other determinants such as poverty, marital misconduct, and problems with in-laws. *“The deceased woman and her family (including the husband and two children [girls at ages 3 and 7]) lived in an encroached land area in the wood, which is very difficult for us to find. People nearby were not aware of the existence of the house until the death. The house was a single room built with mud and woods. They were having numerous economic issues”* (C9: Field Note).

Here, social isolation was interpreted as a result of the tarnished personality of the woman. The narratives of the relatives revealed a strong negative impression of her. *“We didn’t have much contact with them because my brother eloped against our blessings. They stayed at my house for about three months. In the meantime, she tried to have an affair with my husband. As a result, we did not associate with her”* (C9: R29). Interviews indicated that the family did not have any membership in social organizations in the area.

The analysis of maternal deaths, C2, C8, C9, and C14, exposed how social isolation increases the risk of maternal death due to a lack of cohesion and social support. Social isolation made it difficult for even the PHM to conduct her domiciliary visits to the isolated woman (C9) as she lived in the jungle. Also, C2, C8, C14 were related to the domestic violence.

Death C8 disclosed how family rejection led to the suicidal contemplation of the deceased woman. Instead of enjoying the positive and nurturing feeling of motherhood, these women were rendered helpless in the face of physical and verbal abuse as evident under the theme ‘Domestic violence’ below. Statement of C8 (R27) indicates that the husband and his parents were insensitive to the risks of a pregnancy. *“After a heated argument with our mother, she was kicked out of the house. They had built a house on our mother’s land. But mother had asked her to leave the premises…” (C8: R25).*

#### Intermediary determinants leading to maternal death

Intermediary determinants are domestic violence, physical or sexual abuse, alcohol or substance use of the husband and the presence of mental health problems either in the mother or a family member characterizing the incidents. Community members reported neglect of their health by the deceased woman on several occasions.

##### Violence abuse and substance use

In this study, four deaths, C2, C8, C13, and C14, were delineated as violence with maternal deaths. However, contextual analysis of each case revealed different root causes. The community revealed gruesome incidents of extended sexual abuse.

*“Her husband has a mentally and physically disabled brother. When she was alone in the room, this disabled brother would pay unwelcomed visits to her. Even when they were having sex, her husband would call his disabled brother into their room. He would force her to have intercourse with this brother”* (C14:R40). The profile analysis in this maternal death revealed that the woman was forced to be submissive due to accepting those unconventional coercions by her husband.

We identified contradictory evidence between health system identification of the cause of death as suicide. According to perceptions of some community members and investigators’ observations, the incident reflected a homicide led by extreme physical violence. *“Her husband is exceedingly addicted to drugs. He always fought with his wife. He committed suicide by hanging himself after poisoning his wife.”* (anonymized).

“*He beat my daughter until she lost consciousness, forcibly poisoned her, and killed her*” *“a lady doctor from the hospital told me it was not a suicide; someone has forcibly poured a pesticide liquid into her nasal cavity”* (relatives of a deceased woman).

Another family emphasized a similar experience. The husband of the deceased woman was an illicit substance user. *“He is a drunkard and once pulled my sister’s hair and kicked her to the ground. One day he beat her until her lips bled. Even during the pregnancy, my sister was always beaten up by him”* (C8:R26).

“*He suspected that she was having another affair. I guess because of that, she was beaten to death and hung afterward to mimic a suicide”* (C8: R24). The relatives doubted that the post-mortem report was fabricated. “*The officials who conducted the autopsy informed our relatives that she was murdered and hung. But with the help of the police, the husband was able to falsify the evidence and escape”* (C8: R24).

The family, community, or healthcare networks did not effectively intervene to address this imminent issue – suicide or murder.

##### Neglect of health issues and self-care during pregnancy and postpartum as perceived by the society

This theme emerged based on the perceptions, explanations and comments of neighbors and relatives on a certain behaviors of pregnant and postpartum women associated with caring for their own health. We observed that the society judged the behaviors of women as suboptimal in relation to health within the generally accepted cultural norms of health practices in pregnancy.

*“I think she suffered from fever because she had a bath every night after finishing her household chores. She did not rest even when she was ill. Even in the morning, she went to the hospital, she hand-washed a basin full of clothes. Her health deteriorated on her way to the hospital, and she was sent to the general hospital immediately.“* (C1:R2). “*She did not care about her health”* C1 (R3).

Sometimes the negligence led women to bear risk pregnancies. According to the neighbors, the doctors have stated in her clinical records the risk of childbearing. *“Signed in red stating that it is dangerous to bear children”* (C3: R10). “*She bathed in a tank filled with cold rainwater in the evening and developed chills later. But she never listened to our advice. Also, she vehemently refused to go to the hospital. She was so illogical!“* (C3: R10). “A*lthough she had asthma, she did not care about it and showered in the evening.“*

Hence, the case depicts that poor education, perverseness, and the preservation of false traditional beliefs of pregnant women are significant obstacles to improving maternal health.

##### Presence of mental health problems within families

Three deceased women were known or suggested to have mental health problems as perceived by the family members (C5, C6, C9).

One deceased woman had been on long-term treatment for a psychiatric disorder, and the doctors had warned her of the risks of conceiving a child. According to her husband, it has been stated even in the clinical card. *“She had severe depression and took medicine. I think we couldn’t conceive due to the effect of those drugs .  It was difficult to control her when an episode developed. At such times, she has thrown household items and ran everywhere” (C6: R19).*

Although not diagnosed, some narratives depicted that the pregnant woman may have had symptoms of depression. Sometimes the family members came up with their own perceptions. *“I think she had some mental illness. I did not know she was pregnant. She never told me anything. She hid all the details”* (C9:R28).

Although it was not directly related to maternal death, we observed that in 5/15 families, another family member was either mentally subnormal or had a disorder.

##### Perceived health system deficits

When reaching a health care facility, a pregnant woman has to surpass countless obstacles to receive adequate and appropriate care. The detailed analysis of C1 and C4, and C5, C7, C10, and C15, revealed an association of maternal deaths with primary health care and hospital levels, respectively.

##### Suboptimal risk mitigation at the primary health care level in the field

Lack of coverage quality of care by the PHM was mentioned as a reason for death by relatives. *“It happened after eight months from her delivery. After her cesarean surgery, she had a wound that turned red and inflamed whenever there was a pressure effect on the scar, such as defecating/urinating. Afterward, she developed a fever. We believe that she got infected through this wound.* (C1: R1). According to the field care guidelines in Sri Lanka, the PHM should pay monthly visits to the houses of infants; however, the pregnant woman’s situation has received no attention. Field observation revealed that they were not in a position to recognize the risk of death in such circumstances.

Death of C4 also exemplifies perceived carelessness and unkindness of the PHM to pregnant women. *“Midwife said that she was untidy, and her wounds were not recovered. In the post-mortem, the doctor said that her womb was filled with pus. Some are saying the Midwife was transferred because of this death”* C4 (R14).

In C9, the PHM did not approach the deceased women as they lived in a remote area with a damaged road. C9 (R30) explained this as *“They lived in a forest illegally. Even the midwife did not go because of the damaged road.”*

##### Inadequate attention and less preparedness for deteriorating maternal health at the institution

The narratives of C5, C7, C10, and C15 reflected the direct negligence of authorities at the hospital level on maternal health. According to the perceptions of the next of kin, inadequate attention to the pregnant woman’s health at the hospital may have contributed to the death. Characteristically, these families did not have significant social problems and were having a higher socioeconomic position.

*“Her ankle got slightly wounded because of a metal hook on her bedframe. Within 48 hours, the wound got bigger. After admitting her to the hospital, they bandaged it and left her on the bed. I asked about the wound doctors. Then they said, ‘she is not the only patient in the hospital, don’t worry, we will cure her. If you want, you can take her to another place. Nurses and doctors were pettish. Eventually, the wound got worse. So she was transferred to the ‘wound ward’* [probably to the surgical unit]*. I got discharged her forcibly and admitted her to hospital B.  After the death, the post-mortem mentioned that products of the aborted fetus remained in her womb, which was deadly. I directly accuse the doctors, and the fault is with the doctors”* (C5: R17).

Some narratives revealed the scarcity of medications and resources to tackle a medical emergency leading to purchasing medicine outside at a higher price. The maternal death investigations indicate an influenza epidemic in the country. However, the verbatim reflects the different types of stress and strain exerted on people due to suboptimal health care practices. *“She only had a fever and cold. She stayed for six days in the hospital, but the illness got severe. Then I asked the doctor to discharge her to take her to hospital B. But the doctor refused it. Afterward, she was transferred to Base hospital C, and I went there. They gave me a prescription mentioning a medicine for her. The intensive care unit (ICU) was full, so she had to wait until another patient in the ICU was transferred. Then she was taken in.” When she was taken to the ICU, she couldn’t breathe. She stayed in the ICU for 14 days and passed away. There were no facilities. Even the blood tests were performed in private laboratories. I spent nearly two lakhs (equivalent to USD 1,311.82) for those 17 days.* (C10: R31).

According to some family members, inattention and negligence of both private and public hospital authorities led to delayed diagnosis. It could be interpreted as lapses in the referral system at the hospital. *“The next morning, my son called me and said she was vomiting again. We took her to a private hospital, but there was no admitting doctor to admit her. Another doctor told us to go to the general hospital… on the day she was admitted, they gave her saline* [intra venous fluids] *for 24 hours. She complained that the pain was excruciating during the needle prick and the cannula site turned bluish. On the day she was discharged, she developed a fever that lasted for about two days… we took her to a private channeling center, and the doctor advised us to admit her again to the general hospital. But she was admitted to a different ward, not the doctor’s ward at the channeling center. Because of that, the doctors in the admitted ward ignored her. Normally, when we admit someone to the hospital, they get x-rays, ECGs, or at least an Echo. At least they could have tried one of these. We took her to a private hospital in Colombo [the capital]. They said they could cure her and reassured us. But suddenly, she was transferred to a nearby teaching hospital without taking any responsibility”* (C15: R42). The case implies specific, notable gaps such as the degree of difference in public and private health services and failures in coordination when caring for and transferring patients.

#### Patterns of social determinants of health leading to specific causes of maternal deaths

**Fig. 1:**
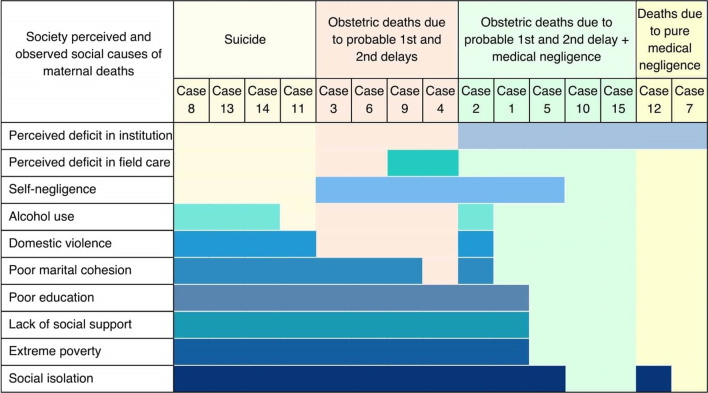
Mapping of the society’s perceived and observed social causes of maternal deaths

**Fig. 2:**
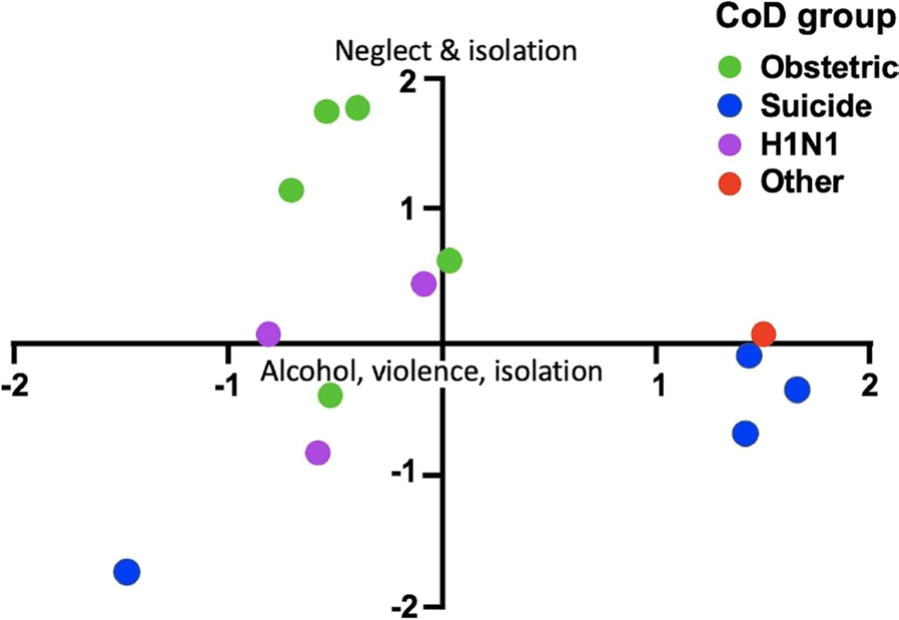
Clustering of social determinants perceived by society on types of maternal deaths. CoD group; Type of maternal death grouped according to cause of death

We observed specific patterns of social determinants leading to delays in health-seeking and types of maternal deaths. Figure 1 presents the mapping of the participant-reported underlying causes and type of death identified through thematic analysis highlighting the clustering of specific reasons for the patterns observed concerning the cause of death. Figure 2 represents the PCA results indicating the social factors leading to types of maternal deaths. The factors identified in the qualitative thematic analysis were included in as social factors in PCA.

**Fig. 3:**
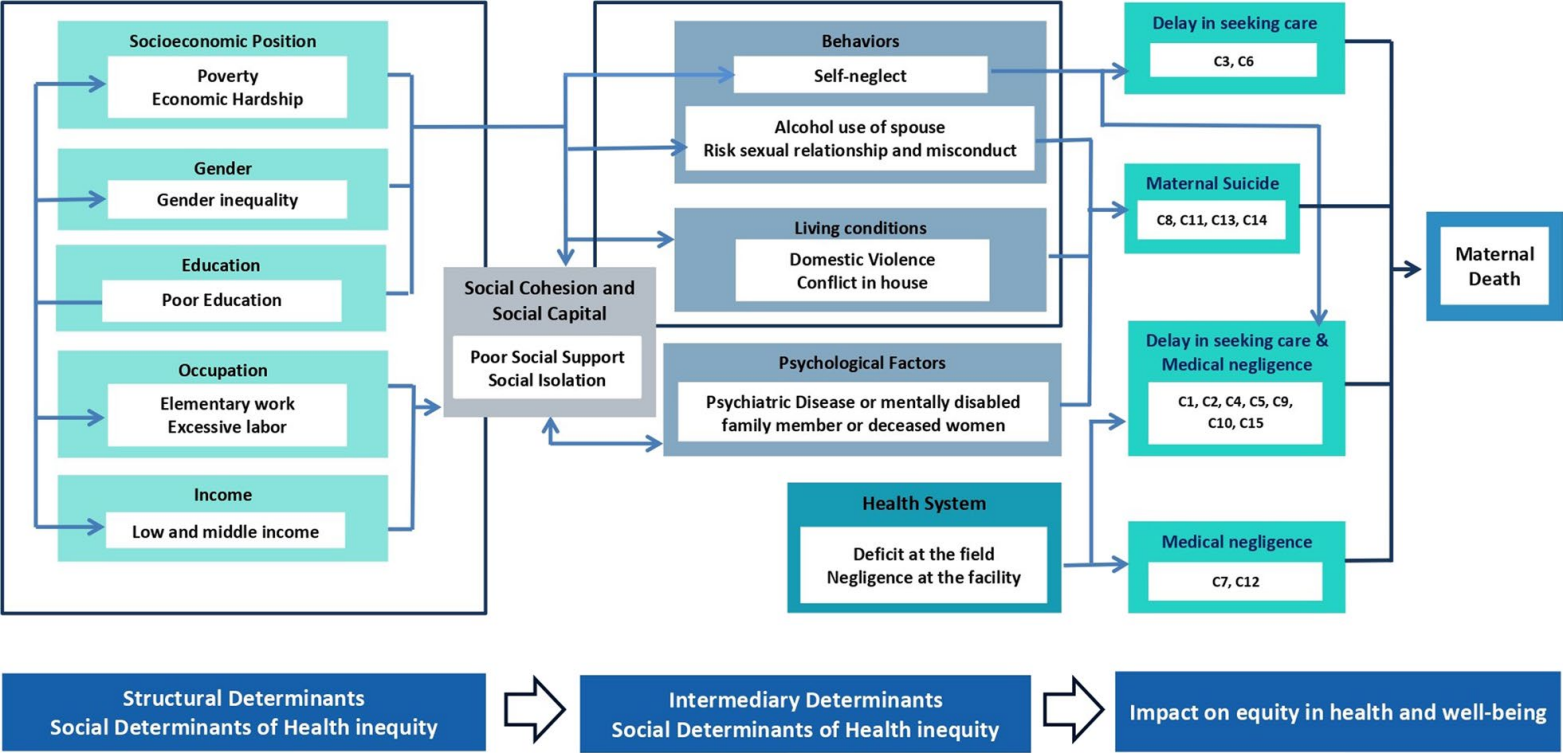
Interaction of the causes of maternal death within the framework of social determinants of health inequities

Of the families that encountered a maternal death, 80% (n = 12) reported social isolation. Clustering the three factors, i.e., social isolation, lack of social support, and extreme poverty, were identified in 66.6% (n = 10) of maternal deaths. This trio of determinants, combined with neglect of own health, led 40% (n = 6) of women to delay health-seeking. A combined cluster of six social determinants, namely poverty, poor education of deceased woman or family, social isolation, lack of social support, domestic violence or abuse, and usage of alcohol or other illicit substances by the spouse, was present in 33.3% (n = 5) cases, of which four were maternal suicides. Marital instability or sexual misconduct was characteristic in 3/4 of suicidal deaths and another four maternal deaths (53% n = 8). Health system deficits were the reason for maternal deaths in three families with a reasonable social status, economy, and education. Mapping the causes and performing psychometric analysis represent these specific patterns visually (Figs. 1, 2, 3).

## Discussion

This study explores social determinants of maternal deaths using a social autopsy approach in rural Sri Lanka. Our approach goes beyond identifying the three delays [17] and health system deficits, which is the focus of routine maternal death investigations. Social causes manifested in 12 out of 15 maternal deaths constructing the roots and paths for the disadvantaged women. Individual structural determinants namely extreme poverty, low educational level, gender inequity, and elementary or below-level occupations of the husband were characteristic to most deceased families. Social isolation which was a result of many structural determinants, was the commonest leading cause and acted through poor social support in constructing the path to maternal deaths. We hypothesized that social risk factors predict certain types of maternal deaths and delays. Poverty, social isolation, and poor social support acted together with alcohol usage, and violence leading to suicides. The same determinants mediating through neglected self-health care led to delay in health-seeking. We also observed the deficits in quality of care at health institutions and the field. The study creates the opportunity to identify the real-life social factors and their patterns, leaving the ground to time test them and utilized as “social risk factors” to identify and act to end preventable maternal deaths.

We identified social isolation and family rejection, with feelings of abandonment and anxiety as a callous toxic component in pregnancy and underpinning maternal deaths. This finding is scarcely reported in high-income countries [42], probably because social isolation has not been commonly considered a determinant of pregnancy outcomes. However, poor social capital has been identified as a determinant of maternal health outcomes in Sri Lanka [43] and a cause of maternal deaths in South East Asia [44].

Inadequate family and social support were evident in these maternal deaths. Musyimi et al. (2020) revealed that social isolation, gender inequity, lacking affection and encouragement in micro-community, and social stigma lead to suicidal ideation in pregnant women [45]. Our study revealed various multi-fabric origins of poor support that worsen the risk of death of women. It is well understood that social support could work as a buffer in preventing mental health problems at global level [46] as well as in Sri Lanka [47]. We highlight the importance of systematically identifying families with poor social support in the rural communities. These families face an overly burden due to poor socio-economic position as well as lack of social support and are at very high risk of developing mental health problems, indicating the need of targeted interventions.

Moving beyond mere identification as risk factors for maternal deaths [48], we observed that structural determinants such as poverty, low educational status, and elementary occupation levels had forced families towards social isolation, creating a synergistic effect to obtain minimal or no support from society. However, this study did not reflect any discrimination due to cast, ethnicity, racism, culture, or religion-related to maternal deaths as reported elsewhere in the world [49].

Poor health literacy of expectant mothers and their families is reported as a root cause of maternal death [44, 50]. Our social autopsy revealed that pregnant women with pre-existing mental disorders and other chronic diseases failed to conceive health risks due to illiteracy. They were not aware of the circumstances due to illiteracy or ignored danger signs regarding an impending calamity/life threat, which might have alerted them to solicit proper medical care at the earliest time. We found no mechanism in convincing illiterate women of the death risk of getting conceived against medical advice.

Many intermediary determinants such as violence, alcohol abuse, psychological problems, disability, and neglect of reproductive health care pave the path to maternal deaths. During pregnancy, violence against women is an agonizing public health concern that can cause physical and psychological anguish [51]. Obviously, the outcomes may be exceedingly catastrophic in the case of pregnant or postpartum women. Previous studies provide evidence on the association between maternal death and intimate partner violence [45, 52]. The present study identified extended violence (physical, verbal, sexual, and coercive and abuse of power) as leading an immediate cause of committing suicide by pregnant and postpartum women. We observed that the social perception contradicts the reported medical causes of maternal deaths. The health care system failed to recognize and act against violence within the family as a health issue. The cases further emphasize the cooperation with the criminal justice system, social service institutes, and the mental and physical health care systems in ending preventable deaths.

The study identified inattention to reproductive health and neglect of personal health as a common factor among diseased women acting to delay in deciding to seek healthcare. In this connection, two types of inattention were recognized—the poor acknowledgment of traditional beliefs/practices related to pregnant and postpartum women and inadequate acknowledgment of health sector advice on reproductive health matters as perceived by the society. The scenarios implicate that maternal health promotion should be more efficiently and systematically be targeted on vulnerable women.

The present study emphasizes that neither the current private health services nor the public health system can effectively dispense the broader public health services required by pregnant women. Kalter et al. confirm that many women in developing countries who seek care never receive effective treatment [18]. Health care reform requires publicizing in a successfully coordinated and collaborative approach, especially to implement measures to identify higher-risk women earlier and monitor them even after childbirth and abortions. Most importantly, social determinants as a part of a public health risk assessment need to be included in the health system to achieve the desired goals related to maternal health in sustainable development goals.

In Sri Lanka, public health midwives play a major role in risk identification, mitigation, and health promotion, which leads to the reduction of maternal deaths [20]. However, the routine training focuses on medical risk factors needed to reduce maternal deaths in the early phases of obstetric transition. However, after achieving low maternal mortality, expansion of the risk factor identification beyond the biomedical model is required, especially in a setting where suicide is the leading cause of maternal deaths [27]. The PHMs are not well empowered to identify and mitigate psychosocial risk factors that act at the early stages of the pathway. Therefore, educating PHMs to recognize households with psychosocial risk factors and bring women at risk for care may be lifesaving.

### Strengths and limitations of the study

One of the main strengths of the present study is that we were able to include 15 out of 18 maternal deaths occurred in the District within two years which is almost close to the population. Emphasis was given to a social autopsy approach where social science researchers approached the participants. Hence the study explored the social factors that are not a routine concern of the national maternal death investigation revealing the importance of a multisectoral approach to obtain a better yield in these procedures. The data analysis and interpretation was conducted by both social science and public health researches indicating careful and rigorous procedures to safeguard the credibility and validity of data.

However, with the above mentioned strengths, the findings of the present study need to be understood with the following limitations. First, even though we collected data on maternal deaths that occurred in the recent past the possibility of recall bias could not be eliminated. However, conducting interviews immediately after bereavement would be unethical due to psychological reasons. Although we took measures to improve credibility of data, the information revealed may not be completely accurate due to the adopting behaviors of the respondents as they noticed the voice recordings and note taking during the interviews. Further, even though we planned to conduct three interviews per case, we were unable to cover three interviews for each case due to the unavailability and the unwillingness to participate in the interviews. Many of the individual structural determinants can have the suggested causal relationship as indicated in Fig. 3. However, as this study is conducted at one time point and due to the qualitative nature, we can only make hypotheses of the causal relationships. However, when it comes to the second stage where intermediary factors govern the hypothesized causal pathways, they are more apparent as the incidents in the in-depth narratives are sorted in a chronological order. Testing these hypotheses using large population based studies would be propitious, never the less difficult as maternal deaths aren’t a common event in the context.

## Conclusion

In rural Sri Lankan settings, diverse social determinants of health, including health system factors, pave the path to maternal deaths. Society perceived psychosocial risk factors are neither captured nor discussed in the routine maternal death investigations, missing the opportunity of identifying probable root causes of maternal deaths. To attain a sustained and long-term declining phase in the trajectory of maternal mortality, cause-specific changes and implementation of a set of strategies such as expanding access to health services, professionalizing midwifery, arousing women, and communities, improving management, and enhancing the ability of the poorest to reach the health services have to be carried out. Public health services and legislative institutions must be socially accountable and transparent with careful record-keeping and public accessibility of records to discard societal misconceptions towards those institutions, which we observed in this study. National and regional leadership could support creative refinements and redesign efforts to amalgamate public and private health programs to ensure the best possible care for pregnant women.

## Data Availability

Supporting and raw data are available upon a reasonable request to the corresponding author.

## Abbreviations

LMICs: Low- and middle-income countries
SDH: Social determinants of health
SDGs: Sustainable Development Goals
ICD-MM: International Classification of Diseases for Maternal Mortality
USD: United States dollar
SRQR: Standards for reporting qualitative research
GCE: General Certificate of Education
ISCO: Standard Classification of Occupations
CoD group: Type of maternal death grouped according to cause of death
PHM: Public health midwife
ICU: Intensive care unit
